# Method of Motion Planning for Digital Twin Navigation and Cutting of Shearer

**DOI:** 10.3390/s24185878

**Published:** 2024-09-10

**Authors:** Bing Miao, Shirong Ge, Yunwang Li, Yinan Guo

**Affiliations:** 1 School of Mechanical and Electrical Engineering, China University of Mining and Technology (Beijing), Beijing 100083, China; 2 Key Laboratory of Intelligent Mining Robotics, Ministry of Emergency Management, Beijing 100083, China; 3 China Academy of Safety Science and Technology, Beijing 100012, China

**Keywords:** shearer, motion planning, digital twin, reinforcement learning

## Abstract

To further enhance the intelligence level of coal mining faces and achieve the autonomous derivation, learning, and optimization of shearer navigation cutting, this paper proposes the methods of shearer digital twin navigation cutting motion planning based on the concept of shearer autonomous navigation cutting technology and intelligent coal mining face digital twins. This study includes the digital twin theory and the construction method of the shearer digital twin navigation cutting motion planning system based on this theory. Based on the digital twin theory, a shearer digital twin navigation cutting motion planning system was constructed. This system supports the service functions of the shearer cutting digital twin, dynamic navigation map digital twin, reinforcement learning environment construction, and motion planning through the physical perception layer, comprehensive data layer, and digital–model fusion analysis layer. Finally, by comparing the effects of the DQN-NAF and DDPG deep reinforcement learning algorithms in the shearer motion planning task within the constructed digital twin environment, the results show that the DQN-NAF algorithm demonstrates better performance and stability in solving the shearer digital twin motion planning task.

## 1. Introduction

Coal is currently the main energy source in China. The intelligent construction of coal mines is of great significance for promoting the transformation and upgrading of the coal industry and achieving high-quality development [1,2]. As of the end of April this year, 1922 intelligent coal mining faces and 2154 intelligent tunneling faces have been built nationwide. The National Energy Administration issued the “Notice on Further Accelerating the Intelligent Construction of Coal Mines to Promote High-Quality Development of Coal,” which requires large coal mines to accelerate the full intelligentization of mining systems. At the same time, the “Guidelines for the Construction of the Coal Mine Intelligent Standard System” propose the establishment of digital twin system standards, covering reference architecture, information models, equipment models, data interfaces, and digital twin service applications for entire mines.

Intelligent coal mines achieve comprehensive intelligent operations by deeply integrating technologies such as artificial intelligence, the industrial internet, cloud computing, big data, robotics, and smart equipment with modern coal mining technologies. This intelligent system has the following key features:

Comprehensive sensing: through a sensor network, intelligent coal mines can monitor multiple dimensions of information in real time, including the mine environment, equipment operating status, and personnel locations.

Real-time interconnection: the introduction of the industrial internet allows various devices, systems, and personnel in the coal mine to be interconnected in real time.

Analytical decision-making: based on big data and cloud computing technologies, intelligent coal mines can rapidly process and deeply analyze the massive amounts of data collected, thus supporting intelligent decision-making.

Autonomous learning: the intelligent coal mine system possesses autonomous learning capabilities, enabling it to gradually learn the operation patterns and anomalies of the mine by analyzing historical data, thereby optimizing operation strategies and preventing potential risks.

Dynamic prediction: through real-time data analysis and modeling, the intelligent coal mine system can dynamically predict the future conditions of the mine.

Collaborative control: in intelligent coal mines, various subsystems can achieve coordinated control.

**Figure 1:**
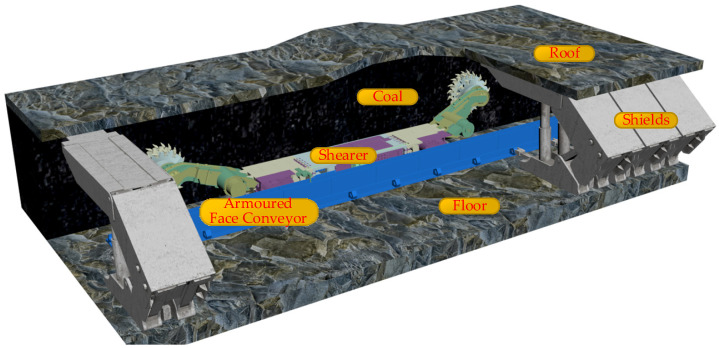
The coal mining workface scenario.

The working scenario of the coal mining workface is shown in Figure 1. The shearer is the most important production equipment in coal mines. The implementation of digital twin navigation and cutting motion planning not only automates the shearer’s operations but also enhances the intelligence of the coal mining process.

The coal mine environment is complex and variable. Geological conditions, ore body structures, and various disturbances (such as vibrations and dust) generated during the mining process can all affect the navigation and cutting motion planning of the shearer. The shearer generates large amounts of data during operation, which need to be collected, transmitted, and processed in real time. This requires the system to have an extremely high computing power and data processing speed to ensure the digital twin model is updated and accurate in real time. To cope with the complex underground environment, the shearer’s cutting motion planning needs to be highly intelligent and adaptive. This necessitates the development of more sophisticated artificial intelligence algorithms to achieve autonomous learning and real-time adjustments.

Through digital twin technology, the shearer’s virtual model can accurately reflect the operational status and environment of its physical entity in real time. Engineers can use this virtual model to precisely plan and optimize the shearer’s cutting motion. By simulating different motion paths and cutting strategies, the best plan can be identified, thereby improving mining efficiency. Through autonomous learning and reinforcement learning in the shearer’s cutting motion planning, the system can dynamically adjust the motion paths and operational strategies based on real-time data, thus enhancing mining efficiency and safety.

The development of digital twins can be divided into three stages. The first stage is the concept proposal. In 2002, Professor Michael Grieves [3] from the University of Michigan first proposed the concept of the digital twin in a Product Lifecycle Management (PLM) course. At that time, industrial software such as PLM and simulation had gradually matured, laying the foundation for building digital twins in virtual space.

The second stage is the application period in aerospace. From 2011 to 2012, a series of outlooks on the application of digital twins in aircraft were released by NASA and AFRL (Air Force Research Laboratory). Digital twins began to be applied early in the aerospace industry. In 2013, Dr. Mark T. Maybury, the Chief Scientist of the U.S. Air Force, conducted a research survey on global future technology trends and summarized the report “Global Horizons”, viewing digital thread and digital twins as game-changing opportunities [4]. NASA hoped that by 2025 each aircraft delivered to the U.S. military would be accompanied by an as-built digital model, allowing for the estimation of lifespan and reliability in virtual scenarios. NASA defined the digital twin of an aircraft as an as-built model or system that integrates multi-physics, multi-scale, and probabilistic simulations. The as-built model itself should be tightly coupled with external shapes, internal structures, and computational fluid dynamics models and be able to map the physical aircraft through sensor data and flight history data [5].

The third stage is the multi-industry expansion application period. Currently, the application of digital twins has evolved from the aerospace field to multiple industries. Industrial enterprises represented by GE and Siemens are accelerating the construction of digital twin solutions to provide innovative enabling services for industrial enterprises. The rapid development of digital twins is closely related to the rise of new-generation information technology and the widespread application of the industrial internet in multiple industries [6]. In the future, the application of digital twins in the industrial field will continue to deepen, accelerating the digital transformation of industrial enterprises. Digital twins are an important component of the Industry 4.0 digital process.

In 2014, Shirong Ge, while leading a National Key Basic Research Development Program “973” project, first proposed an innovative concept: an autonomous navigation cutting technology for shearers based on a coal seam Geographic Information System (GIS).

This technology marked a new concept in the development of shearer cutting technology [7]. It utilizes detailed coal seam detection maps for planning cutting movements and adopts navigation control technology to achieve unmanned shearer cutting operations. This effectively addresses the challenges brought by coal seam variations, allowing the real-time adjustment of cutting paths and ensuring the autonomy of operations. By 2020, Shi-Rong Ge further proposed the concept of the Digital Twin Smart Mining Workface (DTSMW) [8]. This is a highly realistic three-dimensional mirror scenario of the mining workface with strong data visualization, human–machine interaction, and full-process self-optimization. The system achieves bidirectional communication and information exchange between the digital twin and the physical entity, comprehensively perceiving the physical space of the unmanned fully mechanized mining workface, monitoring the production process and performance in real time, and providing three-dimensional visual reproduction of virtual scenarios. This significantly enhances the intelligence level of the fully mechanized mining workface.

In the domain of predictive research based on digital twins, Xu et al. [9] and Renganathan et al. [10] introduced a fault diagnosis framework that integrates digital twins with deep transfer strategies and another framework that incorporates aerodynamic data. To address the challenge of high simulation complexity, Magargle et al. [11] used multidisciplinary simulation methods and developed reduced-order models to create a simulation-based digital twin model. Fotland et al. [12] employed the Arbitrary Lagrangian–Eulerian Absolute Node Coordinate Formulation (ALE-ANCF) method to significantly enhance computational efficiency and accuracy. Guivarch et al. [13] proposed developing digital twins using multibody simulation technology, improving computational efficiency by simplifying rotor components. To balance computational efficiency and accuracy, Kapteyn et al. [14] combined degradation model libraries with Bayesian state estimation to construct a digital twin-driven structural model, thus enhancing the reliability and precision of digital twins. Ganguli et al. [15] established a digital twin for a single-degree-of-freedom dynamic system, defining it as a dual-time-scale model, and discussed the accuracy of digital twin predictions when system mass and stiffness change simultaneously. In the digital twin of a single-degree-of-freedom dynamic system, Chakraborty et al. [16] applied Gaussian processes to address the uncertainty issues of sparse and noisy data in digital twins. To construct a multi-time-scale digital twin for the degradation process, Adhikari et al. [17] introduced the Mixture of Experts (ME-GP) and Mixture of Experts (MOE) framework, enabling rational predictions for future time steps by identifying key parameters from continuous measurement data, thus offering new methods and insights for solving practical problems.

The construction of digital twin models for intelligent mining equipment is the foundation and a key part of digital twin technology. Therefore, it is necessary to analyze and build digital twin models containing relevant model information for different types of intelligent mining equipment and their specific application requirements [18].

The construction formula for the digital twin of intelligent mining equipment is as follows:

(1) $${\mathrm{MG}}_{\mathrm{DT}}=\left({\mathrm{PE},M}_{\mathrm{DT}},{\mathrm{DB}}_{\mathrm{DT}}\right)$$

MG_DT_ represents the digital twin of intelligent mining equipment, PE (physical entity) represents the physical entity model of intelligent mining equipment, M_DT_ (digital twin model) represents the twin model of intelligent mining equipment, and DB_DT_ (digital twin database) represents the digital twin data model of intelligent mining equipment. The physical entity model PE (physical entity) of intelligent mining equipment refers to the virtual representation of the functions and sensory perception of intelligent mining equipment in the real world.

**Figure 2:**
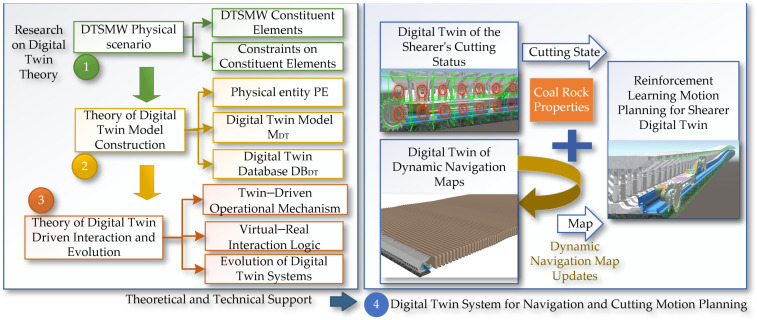
Technical architecture of digital twin navigation and cutting path planning for shearer.

Based on the concepts of shearer autonomous navigation cutting technology and the Digital Twin Smart Mining Workface, this paper proposes the theory and methods for the digital twin navigation cutting motion planning of shearers. Digital twin (DT) technology is used to create virtual models of physical entities, achieving bidirectional mapping, a dynamic interaction, and a real-time connection between the physical world and the digital world. The core content includes digital twin theory, the digital twin of the shearer’s cutting state, the digital twin of the dynamic navigation map, and the key parts of motion planning through deep reinforcement learning based on digital twins. The specific content is shown in Figure 2.

First, the digital twin theory includes three main aspects: the physical scene of the smart mining face, the construction theory of the digital twin model, and the mechanisms of digital twin-driven interaction and system evolution. The research on the physical scene of the intelligent mining face is subdivided into the constituent elements of the intelligent mining face and the constraint relationships of the equipment environment, providing a solid foundation for the construction of the digital twin model. The composition of the digital twin model includes physical entities, twin models, and twin data models. The digital twin-driven interaction and evolution theory is further refined into the digital twin-driven operation mechanism and the logic of the virtual–real interaction.

**Figure 3:**
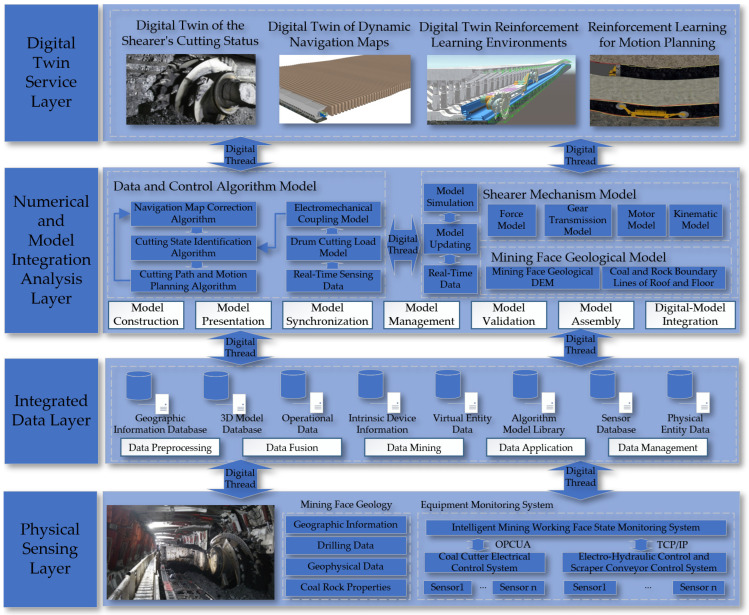
Digital twin navigation cutting motion planning system architecture for the shearer.

Next, supported by the digital twin theory, a digital twin navigation cutting motion planning system for the shearer is constructed, as shown in Figure 3. Firstly, the cutting state of the shearer is digitally twinned by combining the digital twin model with real-time sensor data. Secondly, a finely detailed coal seam navigation map that can be dynamically updated is created to provide real-time, accurate navigation path information. Finally, a reinforcement learning environment based on the digital twin is established to achieve an interaction between the physical and virtual models, and reinforcement learning optimization algorithms are used for the digital twin navigation cutting motion planning of the shearer.

This architecture consists of four layers: the Physical Sensing Layer, the Integrated Data Layer, the Numerical and Model Integration Analysis Layer, and the Digital Twin Service Layer. The system integrates the real-world shearer navigation and cutting process, the digital twin model scenarios, the digital twin data model, and the function-oriented application layer. The digital twin environment replicates the real-world shearer navigation and cutting process into the digital twin operational environment through digital means. By invoking various modules within the system, it achieves adaptive data fusion, intelligent analysis, and optimal planning. Due to the high computational and storage requirements of the digital twin system, it cannot operate on edge devices. To ensure data transmission speed, 5G technology is commonly used, with specific communication protocols including Modbus, OPC/UA, and TCP/IP.

**Figure 4:**
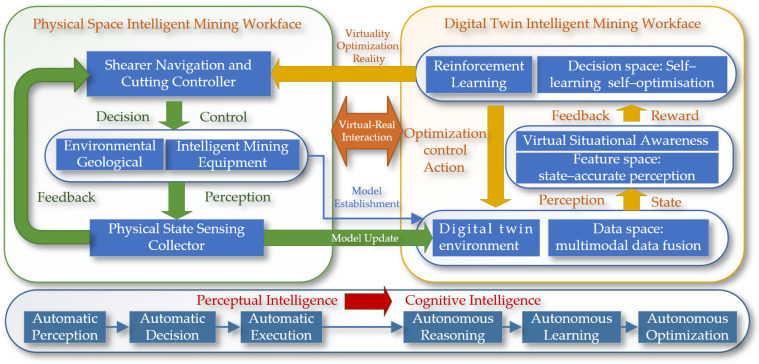
The dual-space interactive logic model.

The digital twin intelligent mining face provides core support for the shearer’s digital twin navigation and cutting motion planning, achieving the transition from perceptual intelligence to cognitive intelligence, as shown in Figure 4. This layer is based on an agent system, equipped with a cognitive intelligence process of “autonomous deduction, autonomous learning, and autonomous optimization”, highlighting the adaptive capability of the agent. In the execution logic of the digital twin intelligent mining face, the shearer agent first utilizes the interactive communication technology of the digital twin to work within the digital twin environment, completing navigation and cutting motion planning tasks, realizing the system’s “autonomous deduction”. To enhance the decision-making performance of the agent, the operation of the digital twin shearer working face is driven by virtual–real mapping technology, providing a simulated real environment to support the training and learning of the agent. Simultaneously, with the help of artificial intelligence algorithms, the “autonomous learning” of the agent is achieved. Finally, to cope with the dynamically changing environment of the shearer working face, the agent system uses intelligent algorithms and the virtual shearer model, through continuous virtual–real interaction, to iteratively optimize decision-making capabilities, enhancing the adaptability and overall performance of the navigation system. This logic layer ensures that the navigation system can effectively adapt to environmental changes, improving operational efficiency and precision.

## 2. Digital Twin of Dynamic Navigation Maps

The key to three-dimensional geological modeling is the representation of geological objects in three-dimensional space. By utilizing various exploration and production data, we can construct initial three-dimensional static geological models that reflect the morphology of geological bodies, providing a fundamental data source for further analysis. However, these initial coal seam models built from static geological data lack precision and cannot effectively provide high-precision geological navigation for the planning of coal mining machine cutting movements.

**Figure 5:**
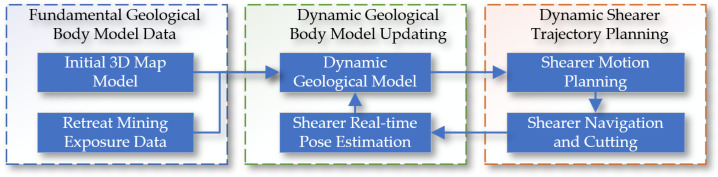
The construction principle of the digital twin dynamic navigation map.

To enhance the precision of the cutting path, we integrate the three-dimensional model data of the coal seam with the cutting trajectory of the coal mining machine. Additionally, we incorporate geological data revealed during the production process, effectively improving the accuracy of the dynamic three-dimensional geological model. Ultimately, we process the generated dynamic three-dimensional geological model into slices. The cutting path is planned based on the maximum undercover amount per operation of the digital twin coal mining machine and the constraints of the production process. Figure 5 illustrates the construction principle of the digital twin dynamic navigation map.

### 2.1. Geological Modeling Data and Modeling Methods

#### 2.1.1. Sources of Model Data

Geological data form the foundation of geological modeling, with their quality and scale determining the effectiveness of the modeling. The sources and characteristics of geological modeling data throughout the entire lifecycle of the coal mining face can be divided into three stages:

Firstly, during the design phase of the workface, data on the distribution of underground coal seams and major geological structures are obtained through surface drilling and three-dimensional seismic exploration. Drilling provides precise but low-density data, whereas seismic exploration offers high-density but relatively lower-precision geological information. Secondly, during the advancement phase of the workface, tunnel construction reveals various geological information such as the thickness, undulations, and structures of the coal seam, which are crucial for establishing a static geological model of the workface. Finally, during the retreat mining phase, the spatial information of the coal seam revealed by the coal mining machine’s cutting and the hidden geological structures within the workface identified through various geophysical methods provide the necessary data support for precise mining and are used to update the three-dimensional geological model. Additionally, geological data obtained using the manual sketching method at the intelligent mining workface are also incorporated into the model updates.

#### 2.1.2. Geological Modeling Methods and Data Model

Three-dimensional geological modeling technology has evolved over the years, summarizing three main modeling methods: the facial model, volumetric model, and mixed model. In this paper, the digital terrain model is implemented based on the Delaunay triangulation algorithm, using a point-by-point insertion method, achieving favorable results.

For the volumetric model, this paper uses a Hybrid Model based on B-REP and CSG technologies, integrating both in the same system to represent entities. Besides describing the surface morphology of geological bodies, it is also necessary to describe the internal attribute information of geological bodies to facilitate spatial analysis and computation. This paper employs the Wireframe-Block method based on the volumetric model to describe the distribution characteristics of internal attributes of geological bodies.

Finally, this paper adopts the Geo3DML standard, also known as the Three-Dimensional Geological Model Data Exchange Format (Geo3DML) (DD2015-06), to unify the data format. This standard was issued by the China Geological Survey to address the issues of sharing and exchanging modeling result data across different departments. It is the only geological three-dimensional model exchange standard in the industry, ensuring that model results are converted according to this standard to enhance service capabilities.

### 2.2. Model Construction

**Figure 6:**
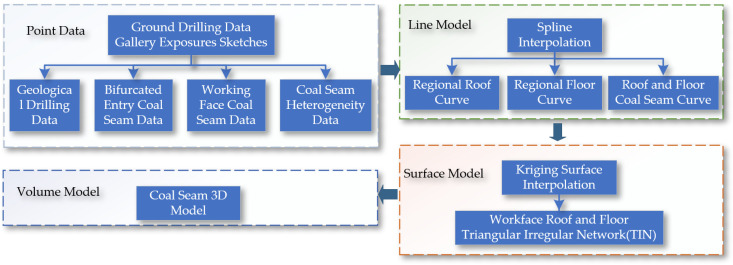
The construction of the initial three-dimensional coal seam.

The construction of the initial three-dimensional coal seam is a process that progresses from points to lines, then to surfaces, and then to volumes, as shown in Figure 6.

**Figure 7:**
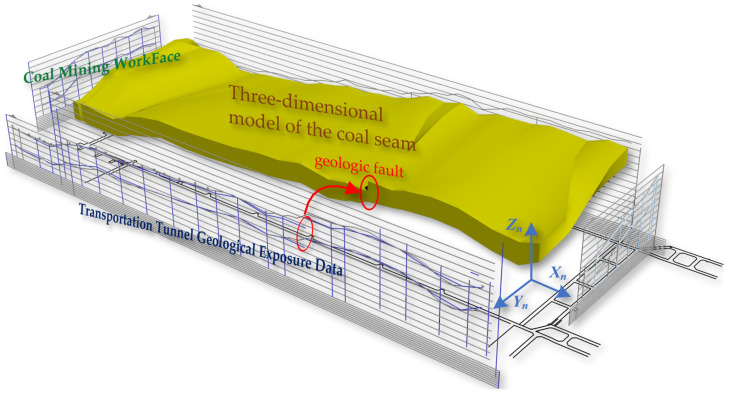
The initial three-dimensional volumetric model of the coal seam.

This is based on data from surface boreholes, gallery exposure sketches, and other sources, which typically include geological drilling data, data from both galleries in coal seams, workface coal seam data, and heterogeneity data of coal seams. Utilizing these point data, line models can be generated through spline curve interpolation methods, such as regional roof curves, regional floor curves, and top and bottom coal seam curves. Subsequently, using the Kriging surface interpolation method, TIN models of the workface roof and floor are created. Ultimately, these data are used to create an initial three-dimensional volumetric model of the coal seam, as shown in Figure 7.

**Figure 8:**
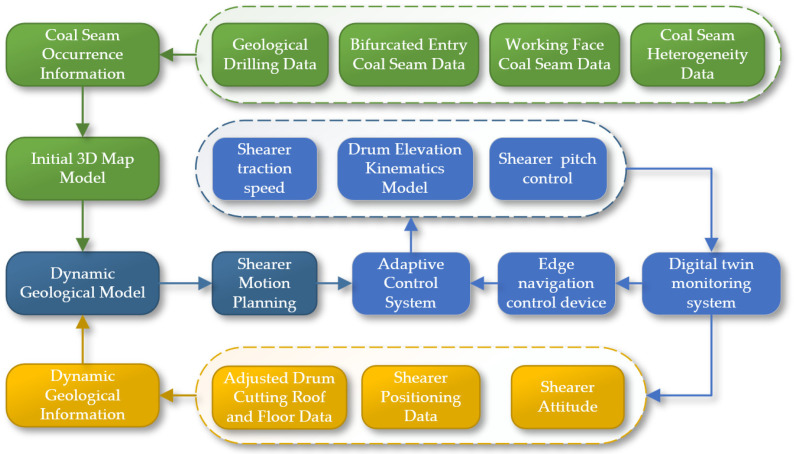
The architecture of constructing a dynamic 3D geological model.

After generating the static 3D geological model, we enhance the accuracy of the 3D geological model by integrating the coal seam’s 3D model data with the shearer’s cutting trajectory using the method shown in Figure 8, thereby constructing a dynamic 3D geological model [19,20]. The cutting trajectory of the shearer’s last cut can be used to verify the accuracy of the 3D coal seam model, with an industrial trial precision of up to 10 cm.

**Figure 9:**
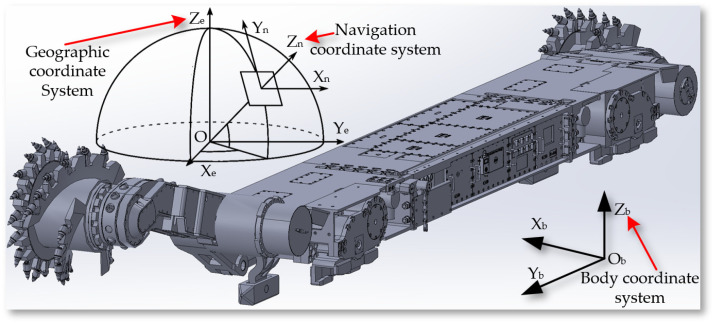
The related coordinate systems of the shearer.

To obtain the cutting trajectory of the shearer, it is necessary to define a three-dimensional coordinate system to describe the position and orientation of points, lines, planes, and objects in space. The related coordinate systems of the shearer are shown in Figure 9. The geographic coordinate system (GCS) O_e_X_e_Y_e_Z_e_ is a latitude and longitude system used to describe points on the Earth’s surface. This coordinate system is based on the Earth’s rotational axis and equator. The navigation coordinate system (NCS) O_n_X_n_Y_n_Z_n_ is used for navigation and positioning, commonly including geomagnetic-based systems and northeast celestial navigation systems. The body coordinate system (BCS) O_b_X_b_Y_b_Z_b_ is fixed at the center of the length of the mining machine’s body and is used to describe the position and orientation of points relative to the body itself. The three key angles describing the mining machine’s posture in three-dimensional space together constitute the Euler Angles.

**Figure 10:**
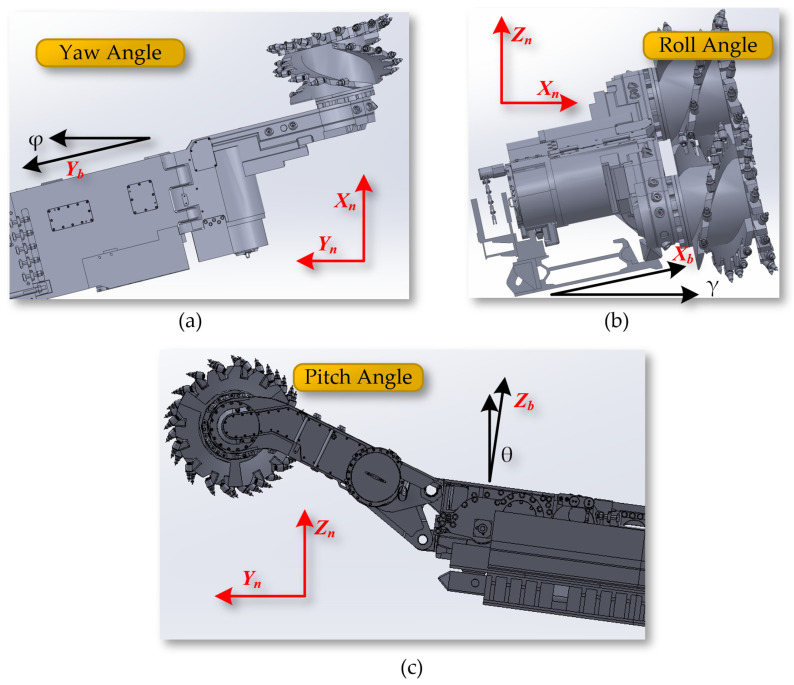
(**a**) The yaw angle; (**b**) the roll angle; (**c**) the pitch angle.

The yaw angle φ indicates the angle of rotation around the Z_b_ axis within the X_b_Y_b_ plane, representing the angle between the Y_b_ axis of the shearer and the northern direction Y_n_. The roll angle γ refers to the angle of rotation around the Y_b_ axis within the X_b_Z_b_ plane, indicating the angle between the X_b_ axis of the shearer and the eastern direction X_n_. The pitch angle θ indicates the angle of rotation around the X_b_ axis within the Y_b_Z_b_ plane, showing the angle between the Z_b_ axis of the shearer and the celestial direction Z_n_, as shown in Figure 10.

The position of the shearer drum is determined by the coordinated movement of the shearer’s height adjustment mechanism. The height adjustment mechanism of the shearer controls the extension of the hydraulic cylinder through a hydraulic servo system, enabling the adjustment of the boom’s height. The boom incline sensor can measure the angle of the boom, but its accuracy is affected by the posture of the shearer. The transmission mechanism of the height adjustment system directly determines the position and height of the cutting drum. By establishing a kinematic model of the height adjustment mechanism, the relationship between the drum height and the length of the height adjustment cylinder can be obtained.

**Figure 11:**
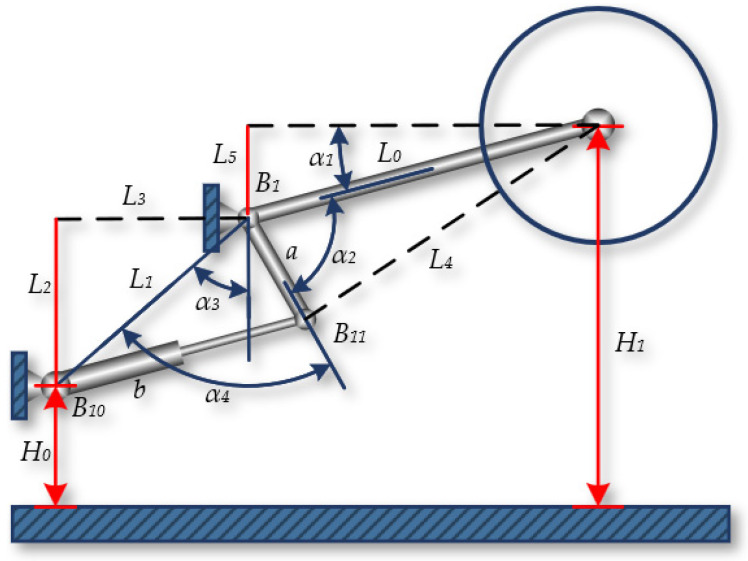
The shearer’s height adjustment mechanism.

The geometric relationship of the shearer’s height adjustment mechanism is shown in Figure 11, and it is known that there is a definite functional relationship between the height of the cutting drum H_1_ and the length b determined by the position B_10_B_11_ after the height adjustment cylinder is extended. According to the geometric relationship shown in the figure, the cutting height H_1_ of the shearer can be calculated by the following formula:

(2) $$\left\{\begin{array}{l} H_{1}=H_{0}+L_{2}+L_{5}\\ L_{5}=L_{0}\sin\alpha_{1}\\ \alpha_{1}=\alpha_{2}+\alpha_{4}-\alpha_{3}-\frac{\pi }{2} \end{array}\right.$$

For the angles α_2_, α_3_, and α_4_, using the known variables a, L_0_, L_1_, L_2_, L_3_, and L_4_ and the single independent variable b, triangles can be constructed. Within these triangles, the arccosine of each internal angle α_2_, α_3_, and α_4_ can be calculated using the cosine law, which relates the lengths of the three sides of a triangle, as shown in the following equation:

(3) $$\left\{\begin{array}{l} \alpha_{2}=\arccos\frac{L_{0}^{2}{+a}^{2}{-L}_{4}^{2}}{{2L}_{0}a}\\ \alpha_{3}=\arccos\frac{L_{1}^{2}{+L}_{2}^{2}{-L}_{3}^{2}}{{2L}_{1}L_{2}}\\ \alpha_{4}=\arccos\frac{L_{1}^{2}{+a}^{2}{-b}^{2}}{{2L}_{1}a} \end{array}\right.$$

Finally, the relationship between the displacement b and the change in the cutting height H_1_ of the shearer can be derived:

(4) $$H_{1}=f\left(a\right)=H_{0}+L_{2}+\;L_{0}\cos\left(\arccos\frac{L_{0}^{2}+a^{2}-L_{4}^{2}}{2L_{0}a}+\arccos\frac{L_{1}^{2}+a^{2}-b^{2}}{2L_{1}a}-\arccos\frac{L_{2}}{L_{1}}\right)$$

### 2.3. Path Planning for Shearer Navigation Cutting

**Figure 12:**
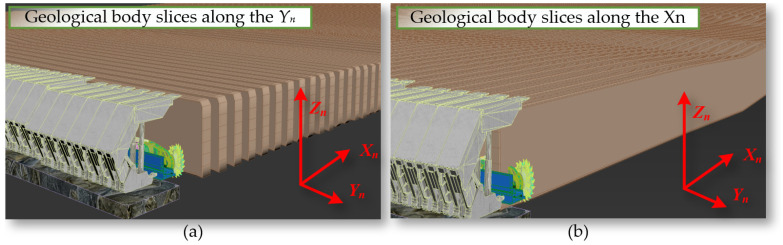
Geological slicing along (**a**) the direction Y_n_ and (**b**) the direction X_n_.

We perform geological slicing on the dynamically generated navigation map along the face direction Y_n_ and the face advance direction X_n_, as shown in Figure 12.

**Figure 13:**
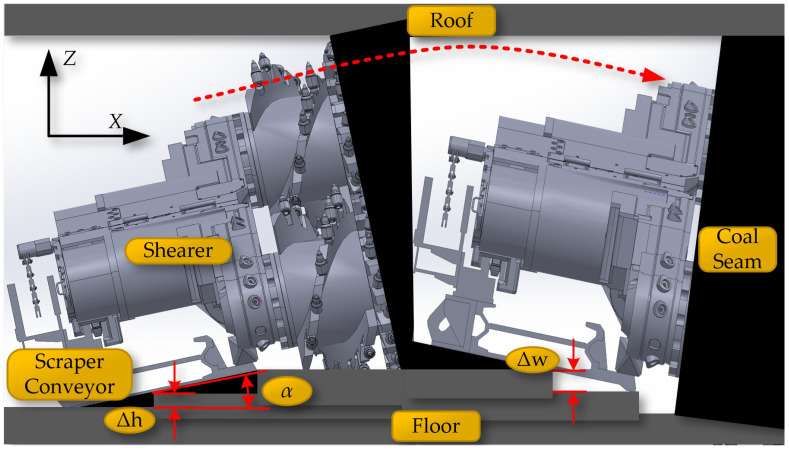
The constraints on the cutting path of the shearer.

Based on the constraints of the shearer’s roll angle (γ), the bottoming amount (Δω), and the coal mining process step amount (Δh), the sliced curves are segmented and linearized, as shown in Figure 13.

**Figure 14:**
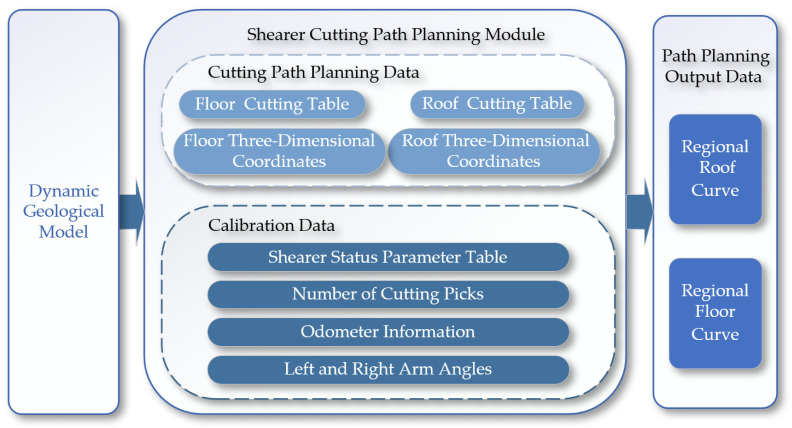
The architecture of the path planning module.

The path planning module shown in Figure 14 controls the swing of the shearer’s boom based on coal seam geographic information to achieve the optimal cutting path for the drum. The module reads the coal seam roof table, coal seam floor table, and inclination tables of the coal seam in two directions. Through an internal algorithm within the cutting motion planning module, it optimizes the carrier coordinate system positions of the roof and floor within the cutting motion plan, generating path curves for both the roof and the floor [21,22].

## 3. Reinforcement Learning Algorithms

Reinforcement learning is a type of machine learning method [23,24]. The training process of reinforcement learning is to find the state–action mapping function that maximizes the expected total reward of the agent [25,26]. Reinforcement learning differs significantly from the widely applied supervised learning. Another important feature of reinforcement learning is the need to balance and trade off the proportions of “exploration” and “exploitation” during the training process [27,28,29].

**Figure 15:**
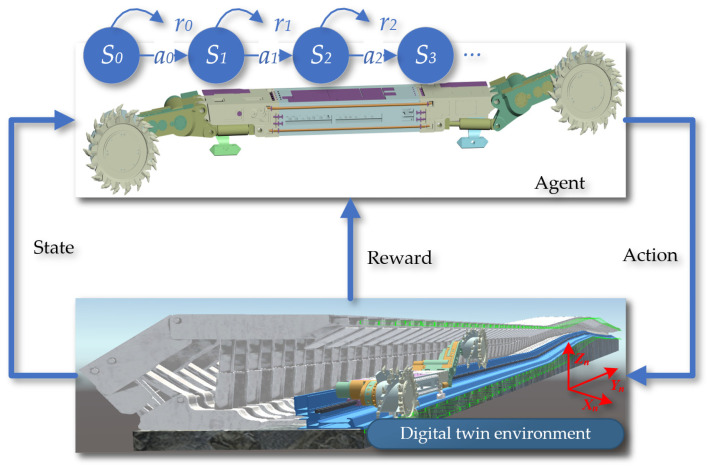
The interaction process between the agent and the environment.

Deep reinforcement learning algorithms used for motion planning have become a research focus in the field of navigation control. Deep reinforcement learning algorithms suitable for shearer navigation and cutting motion planning must be capable of handling multi-dimensional state spaces and continuous outputs. Within the framework of reinforcement learning algorithms, the entity that interacts with the environment and makes decisions is called an agent. The object with which the agent interacts is defined as the environment. In Figure 15, the interaction process between the agent and the environment is displayed, which is key to achieving autonomous learning and decision-making.

**Figure 16:**
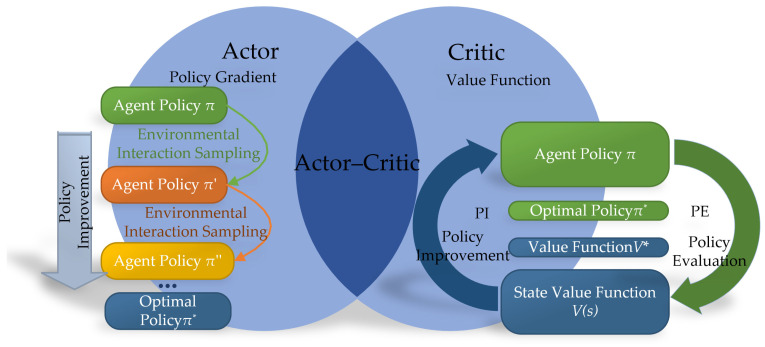
The architecture of the value functions and policy gradients.

From the perspective of algorithmic principles, reinforcement learning can be categorized into methods based on value functions and policy gradients, as well as hybrid strategies that combine these two approaches. These different methods reflect the diversity in the design and implementation of strategies in reinforcement learning, as shown in Figure 16. Algorithms based on value functions mainly focus on evaluating and optimizing the value of each state, thereby indirectly deriving the optimal strategy. In contrast, methods based on policy gradients directly seek to optimize strategies within the policy space, improving expected returns by adjusting policy parameters. The combined approach attempts to utilize the advantages of both strategies to achieve more robust and effective learning outcomes.

In reinforcement learning (RL), an experience tuple typically consists of the four elements s, a, s′, and r, which represent the following:

s (state): The current state. It describes the environmental condition of the agent at a particular moment. In this context, it refers to the three coordinate values of the target positions of the front and rear arms of the coal mining machine, the three coordinate values of the center of the coal mining machine’s body, the height coordinates of the top and bottom plates, and the height coordinates of the front and rear drums.

a (action): The action taken by the agent in state s. The action is the decision made by the agent in a given state, determining what to do next. In the task of motion planning for the coal mining machine, the action space consists of the expected angular changes q of the two arms at the next time step and the increased traction force f of the coal mining machine.

s′ (next state): The next state to which the environment transitions after performing action a. s′ is the result of state s and action a, reflecting the dynamic changes in the environment.

r (reward): The immediate reward obtained after performing action a. The reward is the feedback the agent receives after the environment transitions from state s to state s’ as a result of action a. In tasks such as motion planning for the coal mining machine, the reward includes the penalty/reward value r1 for the distance between the front drum and the top plate and the penalty/reward value r2 for the distance between the rear drum and the bottom plate.

### 3.1. Improved DQN Normalized Advantage Function Algorithm (DQN-NAF)

**Figure 17:**
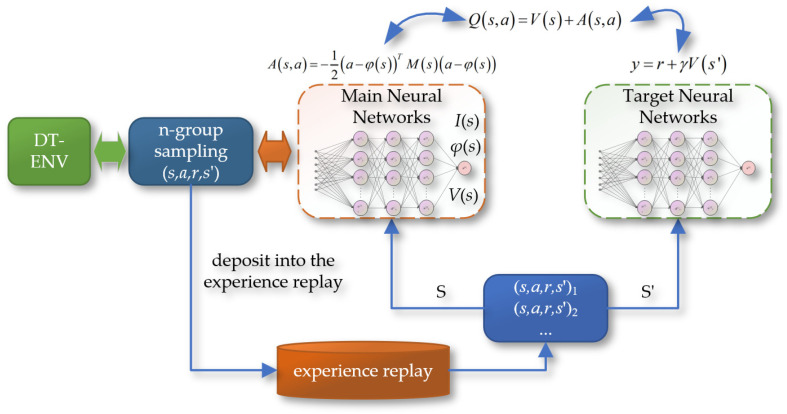
The schematic of the DQN-NAF algorithm.

The Deep Q-Network Normalized Advantage Function (DQN-NAF) algorithm, as shown in Figure 17, involves the design of two neural networks: the implementation network and the target network. The network output includes three main components: the triangular matrix I(s), the action vector φ(s), and the estimated state value function V(s). During training, the DQN-NAF algorithm uses the TD(N) method to update network weights. The implementation network estimates the Q value for each state–action pair by calculating Q(s, a) = V(s) + A(s, a), where A(s, a) is the action advantage function, indicating the degree to which choosing action a is better than the average action. The target network is used to estimate the maximum Q value of the next state V(s′). The loss function is the mean squared error between the predicted Q values and the target Q values. Additionally, the DQN-NAF algorithm includes an experience replay pool, which stores sampling data obtained from interactions with the environment, supporting batch learning and non-online updates, which helps stabilize and improve the efficiency of the learning process.

**Figure 18:**
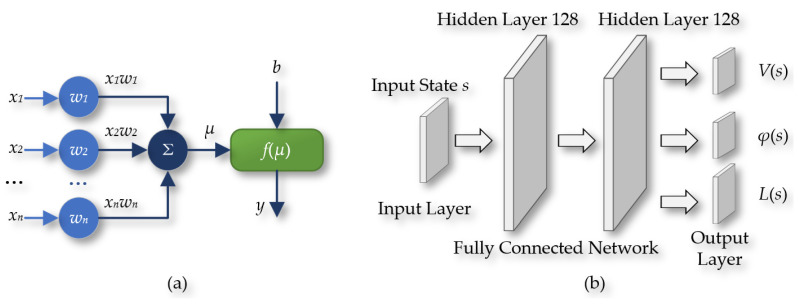
(**a**) The feedforward neural network; (**b**) the network of the DQN-NAF algorithm.

The DQN-NAF algorithm utilizes two sets of structurally identical feedforward neural networks, as shown in Figure 18, namely, the reality neural network and the target neural network. These networks include an input layer, two hidden layers (each containing 128 neurons), and an output layer. The input to the network is the state S of the agent, and the output includes three key components: the state value function V(s), the action vector φ(s), and the column vector L(s) used to construct the diagonal matrix. In this paper, the tanh function is used at the output layer to limit the range of outputs, while the ReLU function is used in the preceding hidden layers.

The implementation details and operational procedure of the DQN-NAF algorithm can be referred to in the pseudocode provided in Algorithm 1.
**Algorithm 1.** DQN-NAF algorithm pseudocode1: Initialize the online Neural Network Q^π^ and Target Neural Network Q^π′^.2: For episode = 1 to M do3:   Initialize action exploration noise ƞ.4:   While the state has not reached a terminal state do5:    Input the state into the online Neural Network Q^π^, select action a = φ(s) + ƞ.6:    Agent performs action a, receives immediate reward r, and the environment state transitions to s′.7:    Store sampled experience (s, a, s′, r) in the experience pool.8:    If the data in the experience pool exceeds the size of the training batch9:      Sample (s, a, s′, r) from the experience pool.10:      Input the action and state into the online Neural Network Q^π^ to obtain the diagonal of triangular matrix I(s) being positive, action vector φ(s), and state value V(s), obtaining $M\left(s\right)=I\left(s\right)I{\left(s\right)}^{T}$, $Q\left(s,a\right)=V\left(s\right)+A\left(s,a\right)$ $A\left(s,a\right)=-\frac{1}{2}{\left(a-\varphi \left(s\right)\right)}^{T}M\left(s\right)\left(a-\varphi \left(s\right)\right)$,11:      Input state s′ into the Target Neural Network Q^π′^ to obtain the value V(s′) and y = r + γV(s′).12:      Use the mean squared error function between Q^π^ and y to update the parameters of the online Neural Network using regression method.13:      With σ being a coefficient less than 1, gradually update the parameters of the online Neural Network towards the Target Neural Network:$\;\;\;\;\;\;Q^{\pi^{\prime }}\leftarrow \sigma Q^{\pi }+\left(1-\sigma \right)Q^{\pi^{\prime }}$14:    End if15:   End while16: End for

### 3.2. Deep Deterministic Policy Gradient Algorithm (DDPG)

**Figure 19:**
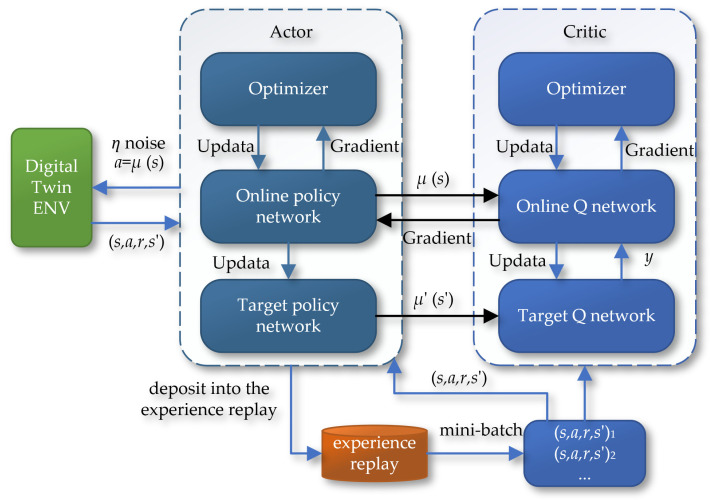
The schematic of the Deep Deterministic Policy Gradient (DDPG) algorithm.

The Deep Deterministic Policy Gradient (DDPG) algorithm further develops on the Actor–Critic architecture by introducing a dual-network structure. This includes the reality network and the target network, which belong to the Actor and Critic modules, respectively. In this setup, the Actor network’s responsibility is to receive input states and produce specific actions, while the Critic network’s task is to evaluate the potential Q values of these actions. The two parts of the network cooperate through continuous training and updates to optimize their performance. Figure 19 details the schematic of the Deep Deterministic Policy Gradient (DDPG) algorithm.

**Figure 20:**
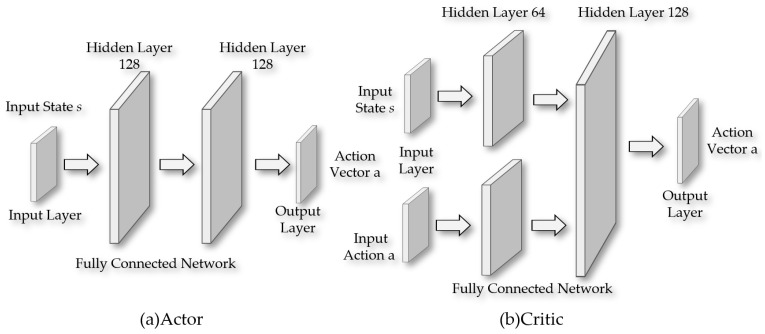
The neural network of the DDPG algorithm: (**a**) the Actor; (**b**) the Critic.

As shown in Figure 20, the Actor part is responsible for generating a deterministic action vector from the input state. In this part of the network, all layers except the output layer use the ReLU activation function, while the output layer employs the tanh activation function to ensure that the range of action outputs is limited to [−1, 1]. The number of neurons in the hidden layers is set to 128. For the Critic part, the agent’s state and actions are fed into two separate first hidden layers, each containing 64 neurons. The outputs of these two layers are then merged and fed into a second hidden layer, which has 128 neurons. The network configuration of the Critic part in terms of activation function selection is the same as that of the Actor, using the ReLU activation function before the output layer and tanh activation function for the output layer itself.

The implementation details and operational procedures of the DDPG algorithm can be referred to in the pseudocode provided in Algorithm 2.
**Algorithm 2.** DDPG algorithm pseudocode1: Initialize online Network parameters θ^Q^ and θ^μ^ for Critic and Actor.2: Initialize Target Network parameters θ^Q′^ = θ^Q^ and θ^μ′^ = θ^μ^ for Critic and Actor.3: For episode = 1 to M do4:   Initialize action exploration noise ƞ, and state s.5:   While the state has not reached a terminal state do6:    Input the state into the Actor’s online Neural Network, select action a = μ(s) + ƞ.7:    The Agent performs the action a, receives immediate reward r, and the environmental state transitions to s′.8:    Store the sampled experience (s, a, s′, r) in the experience pool.9:    If the data in the experience pool exceeds the training batch size10:      Sample (s, a, s′, r) from the experience pool.11:      Use the mean squared error loss of Q(s, a) and the target function $y=r+\gamma Q^{\prime }(s^{\prime },\mu^{\prime }(s^{\prime }))$ to update the parameters of the Critic’s online Neural Network.12:      Use $\nabla J={\left.\frac{1}{N}\Sigma_{i}\nabla \mu \left(s_{i}\right)\nabla Q\left(s_{i},a\right)\right|}_{a=\mu \left(s_{i}\right)}$ to update the Actor’s Target Network.13:     Update the Target Neural Networks of both the Critic and Actor, with σ being a coefficient less than 1: $\{\begin{array}{c} \theta^{Q^{\prime }}\leftarrow \sigma \theta^{Q}+\left(1-\sigma \right)\theta^{Q}\\ \theta^{\mu^{\prime }}\leftarrow \sigma \theta^{\mu }+\left(1-\sigma \right)\theta^{\mu^{\prime }} \end{array}$
14:    End if15:   End while16: End for

## 4. Construction of a Digital Twin Reinforcement Learning Environment

**Figure 21:**
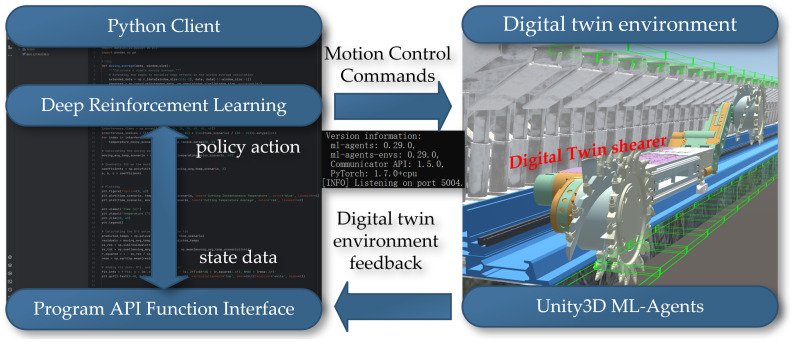
The interaction between RL algorithm and ML-Agents software.

The essence of reinforcement learning lies in the agent’s need to learn through continuous interaction with the environment and a constant process of trial and error, which forms the core training process of reinforcement learning. ML-Agents is an open-source framework developed using Unity3D (2020.3.43 f1c1), specifically designed for training agents in virtual environments to accomplish a variety of tasks. The ML-Agents framework provides a powerful and flexible environment, supporting developers in implementing and testing complex reinforcement learning algorithms in the virtual world, thus further promoting the integration and application of machine learning and virtual simulation technologies. Python (3.11), with the help of the Pytorch software (1.7.0) tool, can quickly set up and complete the neural network computation part; hence, it is widely used in the data analysis and artificial intelligence fields. Using Python to implement deep reinforcement learning algorithms and communicate with the ML-Agents (0.29.0) software for joint simulation is illustrated in Figure 21.

The core of the motion planning method for the mining machine described in this article is the construction of a digital twin environment. The digital twin environment (DTE), compared to the digital twin prototype (DTP), utilizes digital twin technology to construct physical models, behavioral models, and knowledge models. Physical models are built based on geometric models and integrate the physical properties, boundary conditions, load effects, and environmental information of the real physical environment. Behavioral models describe the changes in the behavior of the physical models under different spatiotemporal conditions, influenced by external dynamic scenes and internal operating mechanisms. Knowledge models include basic knowledge models and generative knowledge models. The basic knowledge model is formed based on historical data, expert experience, industry standards, and norms, establishing a set of established and continuously improved knowledge rules. The generative knowledge model updates and adjusts itself over time, mainly relying on machine learning algorithms, through data integration and mining functions to formulate new knowledge rules, enhancing the model’s capabilities in intelligent analysis, prediction, and control.

Using digital twin technology to build reinforced learning environments offers significant advantages as it can more accurately and realistically replicate the conditions of the physical world. Digital twins can integrate real-time data from sensors and other IoT devices, enabling reinforcement learning models to dynamically learn and adapt to environmental changes. This is particularly crucial for applications that require continuous adaptation and optimization. Digital twin technology can create highly detailed and accurate simulation environments that almost perfectly mimic the conditions of the real world. This ensures that reinforcement learning models can interact with a virtual environment whose behavior closely resembles that of the physical environment, thus enhancing the reliability of learning strategies.

**Figure 22:**
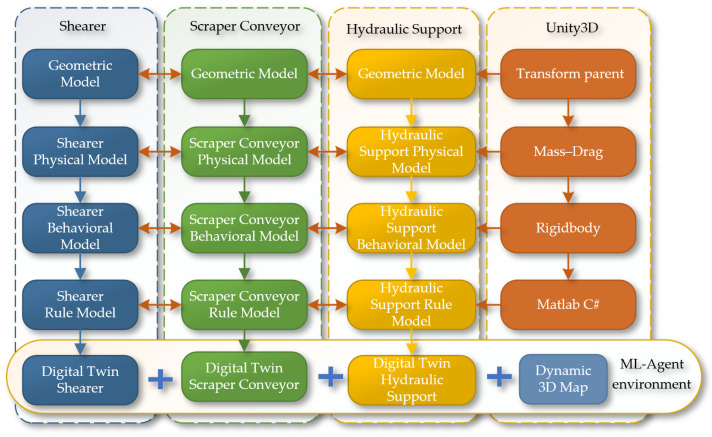
The principles of reinforcement learning environment construction.

In Unity3D, building a three-dimensional coal seam and intelligent mining equipment model for joint analysis digitalizes all elements of the production process, creating a more comprehensive and accurate dynamic map that integrates equipment models into reinforcement learning, which will provide more effective guidance for actual production. Based on the virtual reality engine Unity3D, the digital twin of the shearer, scraper conveyor, and hydraulic support on the three-dimensional coal seam model is implemented to construct a reinforcement learning environment, with the overall research framework shown in Figure 22.

### 4.1. Coal Seam Environment Construction

**Figure 23:**
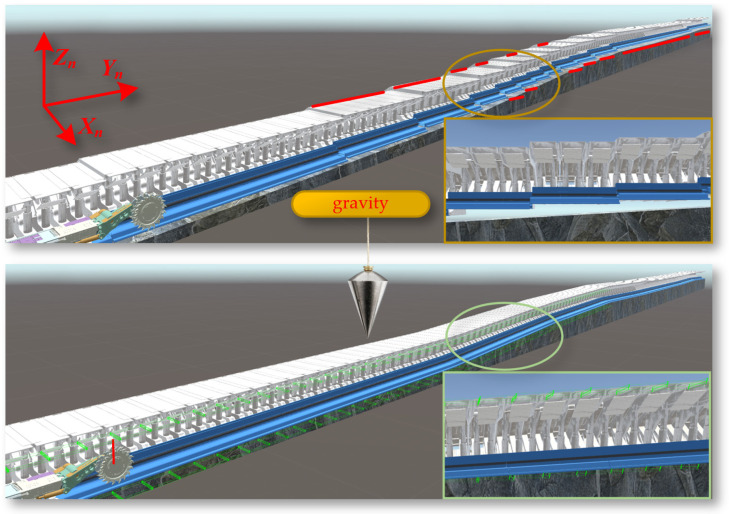
The principle of gravity in physical engine functioning.

First, import the three-dimensional geological body model and intelligent mining equipment model files into the Assets directory of the Unity3D project. In the Project window of Unity3D, locate the imported models and instantiate them by dragging them into the Scene Editor’s Hierarchy window, at which point the models will appear in the scene. Select the model object in the scene, click “Add Component” in the Inspector window, and choose Rigidbody. This component is a core part of the Unity3D physics engine, responsible for handling physical properties such as acceleration, mass, and damping. Activate the “Use Gravity” property of the Rigidbody component and adjust parameters such as Mass and Drag to achieve the desired physical effects. As shown in Figure 23, the digital twin shearer, scraper conveyor, and hydraulic support move downward under the influence of gravity, and after colliding with the virtual coal seam floor, they adaptively fit onto the complex virtual coal seam bottom.

**Figure 24:**
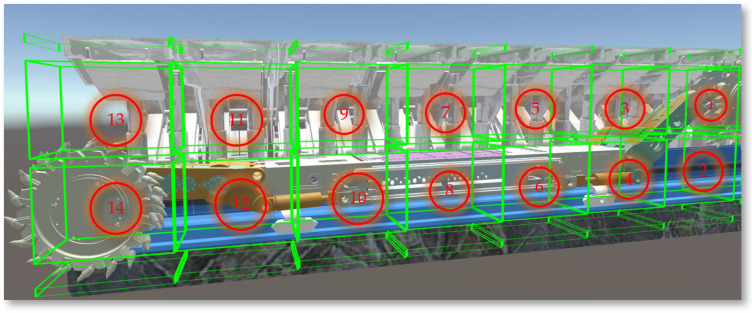
The 3D coal seam model.

After constructing a dynamic and precise three-dimensional coal seam model, it becomes very important to study how the coal seam model can specifically guide the operation of the shearer. Among other things, the three-dimensional coal seam model not only provides a baseplate for equipment layout but also serves another important function: providing data on the coal seam roof and floor that can guide production for the shearer at the current cutting drum. Using a planar function triangulated mesh model would generate a large amount of discrete data points that are difficult to handle. As shown in Figure 24, converting the surface model into a volumetric model allows for the sorting of generated data before storing them in a database. When the shearer reaches the current position, scripts are used to extract data and achieve the extraction of the contour position of the coal seam roof at the intelligent mining face.

**Figure 25:**
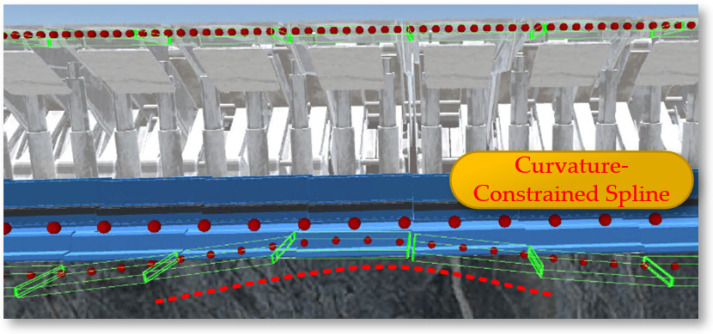
The curve of the coal seam roof at the workface.

After converting to a volumetric model, the drum can be set as spheres bounding spheres, requiring only the storage of the center coordinates and radius. The roof and floor are set with AABB bounding boxes, also requiring only the storage of center coordinates and the radii along the three axes. In intersection tests, calculate the distance between the centers of the spheres; if it is less than the sum of the radii of the two bounding boxes, they are deemed to intersect, which also makes the solution speed of the spherical bounding box the fastest. After Unity3D reads the body coordinates, it uses the LineRenderer component to connect and draw the curve of the coal seam roof at the working face, as shown in Figure 25.

### 4.2. Digital Twin Scene for Shearer

**Figure 26:**
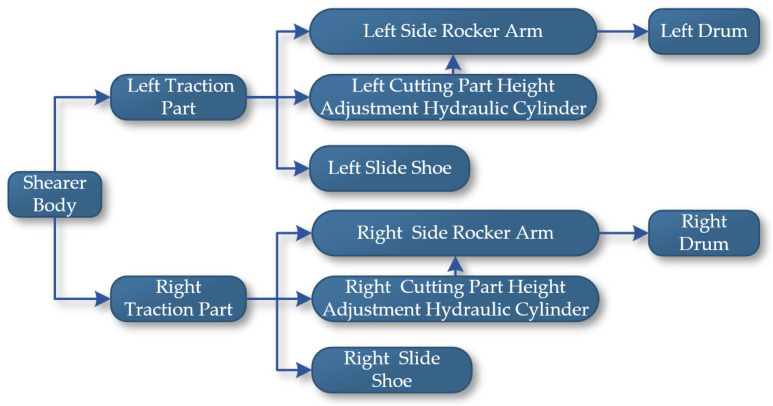
The structural relationships of the shearer.

The Unity3D engine requires the establishment of parent–child relationships for objects composed of multiple structural levels or those with defined motion relationships between them to enable more accurate and efficient operation and control of each level. Declarations of the Transform component for each part include the shearer body, left and right drums, left and right rocker arms, left and right height-adjusting cylinders, left and right slide shoes, etc. By analyzing the main structures and functions of the drum-type shearer, parent–child relationships are established in Unity3D, with definitions for setting the parent of each child object using Transform.SetParent (Transform parent), as shown in Figure 26.

**Figure 27:**
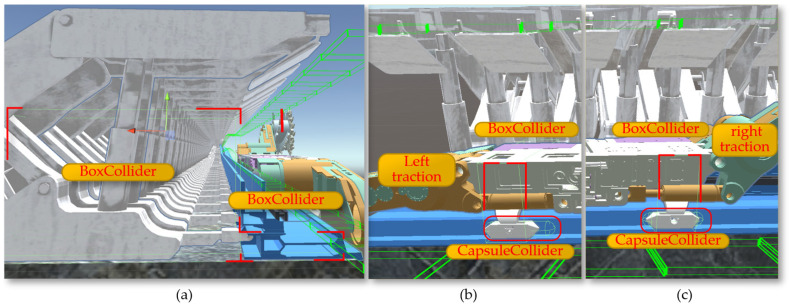
The configuration of the collider: (**a**) the BoxCollider; (**b**) the left CapsuleCollider; (**c**) the right CapsuleCollider.

As shown in Figure 27a, after adding movement scripts to the left and right slide shoes, the shearer can move along the scraper conveyor track. To enable the left and right guide slide shoes and the left and right support slide shoes to move in coordination and further enhance the accuracy of the shearer’s movement in the digital twin environment, additional colliders are added to the shearer. First, a BoxCollider (box-shaped collider) is added to the hydraulic supports and the scraper conveyor track.

By adding a BoxCollider to the left and right guide slide shoes, interference issues between the shearer and the scraper conveyor can occur during the movement of the shearer along the conveyor. As shown in Figure 27b,c, optimizing and adding a CapsuleCollider (capsule-shaped collider) to the left and right support slide shoes ensures that the collider conforms to the external shape of the support slide shoes. This effectively prevents mechanical blockages that could stop movement due to the support slide shoes colliding with the BoxCollider in the middle trough at undulating positions on the baseplate, allowing the defined collider of the shearer to slide through when colliding in the middle trough.

**Figure 28:**
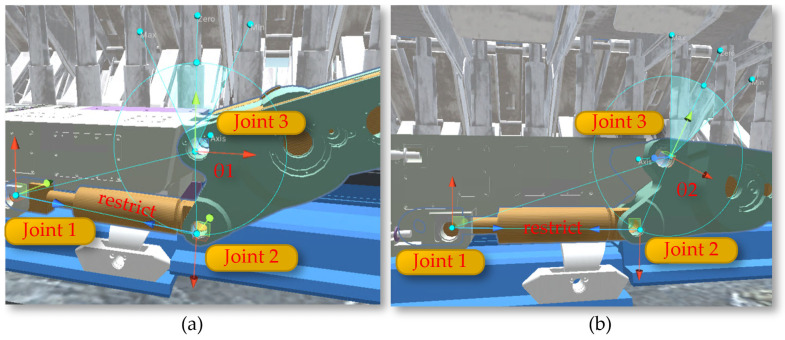
The rotation of the shearer’s drum: (**a**) the angles θ1; (**b**) the angles θ2.

The rotation of the shearer’s drum utilizes the rotation feature of the Rigidbody component in Unity3D, applying a rotation speed to the drum to enable it to rotate and cut the coal wall. The arm height adjustment achieves the rotational movement of the shearer’s arm setting joints Joint1, Joint2, and Joint3 and incorporating the calculation of the shearer arm’s angle adjustment (Equation (3)) into the motion, as illustrated in Figure 28. Through top plate tracking and undercover measurements, the arm adjustment angles θ1 and θ2 are calculated, and the calculated angles are assigned to the arm as the speed of the arm’s rotation, achieving the rotational movement of the arm.

### 4.3. Digital Twin Scene for Hydraulic Supports

**Figure 29:**
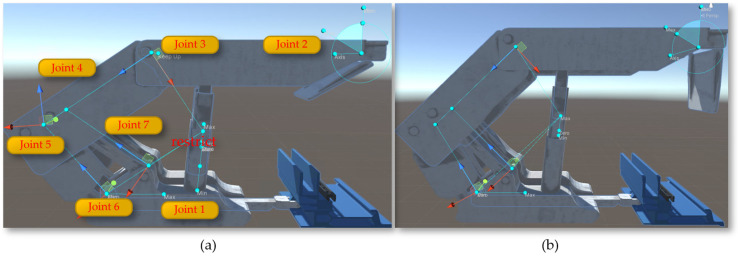
(**a**) The joint configuration of digital twin hydraulic supports; (**b**) the state of the hydraulic supports after being lifted.

Due to the large number of joints in the digital twin hydraulic supports, as shown in Figure 29a, the use of Hinge Joint components combined with the MGS Machinery plugin in Unity3D can bring greater flexibility to the hydraulic supports. Using the MGS Machinery plugin in Unity3D, which facilitates mechanical motion and interaction, a hydraulic.cs script is configured on each hydraulic support to drive the digital twin model. The HydraulicSlideFunction and HydraulicLiftFunction control the shifting and lifting processes of the hydraulic supports, respectively. Under the control of the Rigidbody and collider physics engine and scripts, based on the motion laws of floating linkage mechanisms, the shifting and lifting processes of the hydraulic supports can be achieved. Figure 29b shows the state of the hydraulic supports after being lifted.

### 4.4. Digital Twin Scene for Scraper Conveyor

**Figure 30:**
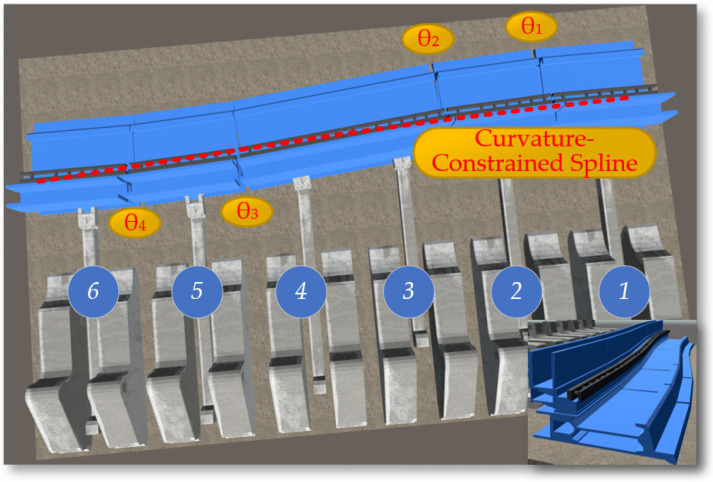
The S-shaped bend of the scraper conveyor.

During the mining process at the intelligent mining face, the scraper conveyor forms an S-shaped bend under the advancement of the hydraulic supports. According to the relevant regulations in the coal industry, when the scraper conveyor is in an S-shape, its horizontal bending angle should range between 1° and 3°, and its vertical deflection angle should range between 3° and 5°. The S-shaped bend of the scraper conveyor is shown in Figure 30.

**Figure 31:**
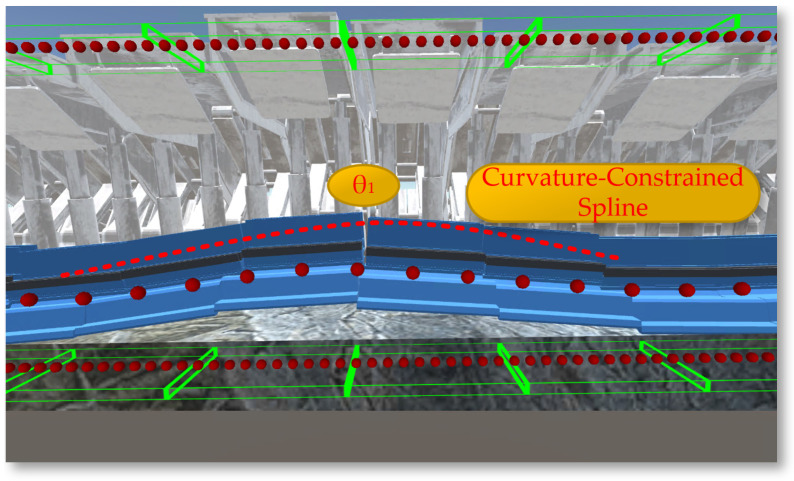
The scraper conveyor varies with the undulation of the coal seam.

In the actual coal mining work environment, the undulation of the coal seam floor is a random and uncertain dynamic environment. In the virtual environment, the digital twin scraper conveyor adapts its posture based on the undulations of the virtual coal seam and the movement constraints between the intelligent mining equipment. As shown in Figure 31, Rigidbody and BoxCollider components are set up in the middle trough of the scraper conveyor, establishing a virtual contact model between the scraper conveyor and the coal seam floor.

## 5. Digital Twin Reinforcement Learning Training

We will explore and compare the control effects of the DQN-NAF and DDPG algorithms in a digital twin environment applied to the motion planning tasks of coal mining machines. Specifically, we will analyze the performance of these two algorithms in controlling the shearer to determine which method is more suitable for such tasks.

**Figure 32:**
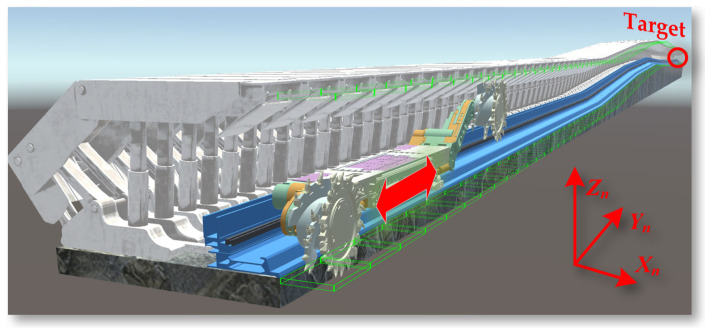
The towing target and cutting task setting of the shearer.

In the task of deep reinforcement learning motion planning for shearer navigation and cutting in the digital twin environment, the reinforcement learning agent controls the digital twin shearer to be towed along the scraper conveyor to reach the target area. The towing and cutting task of the shearer is shown in Figure 32.

**Figure 33:**
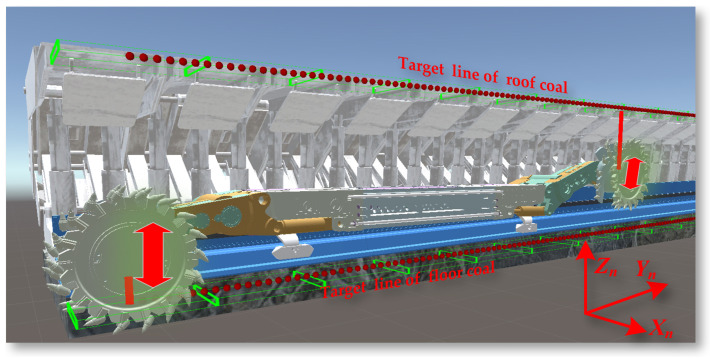
The environment for the shearer drum top and bottom tracking task setting.

In the actual coal mine working environment, the coal seam floor has undulations, and the posture of the shearer itself also changes accordingly. It is necessary to adjust the left and right cutting part cylinders to ensure the edges of the front and rear drums reach within a certain range of the target positions at the top and bottom of the coal seam. The environment for the shearer drum top and bottom tracking task is shown in Figure 33.

**Figure 34:**
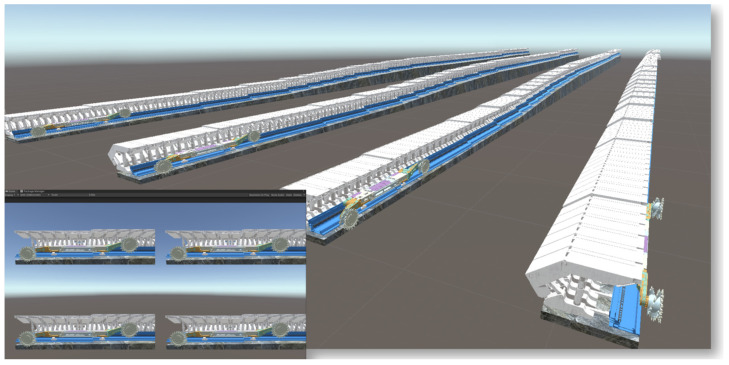
The ML-Agents four-scene parallel accelerated training.

Training was conducted using a 13th Gen Intel(R) Core(TM) i7-13700KF 3.40 GHz processor with an NVIDIA GeForce RTX 3090 discrete graphics card environment created through an Anaconda3 Python environment and ML-Agents software package visualization tool Tensor board 2.11.2. Four scenarios were constructed in ML-Agents, as shown in Figure 34, to accelerate model training.

When designing the input for the reinforcement learning algorithm, we defined a 10-dimensional state vector to describe the current environment. This state vector includes the angles θ_1_ and θ_2_ of the two rocker arms, the three coordinate values of the target position, the three coordinate values of the shearer’s center, the top and bottom plate height coordinates, and the front and rear drum height coordinates. These parameters collectively form the algorithm’s input, providing the agent with comprehensive environmental information to enable effective decision-making and motion planning:

(5) $$s=\left\{\theta_{1},\theta_{2},x_{r},y_{r},z_{r},x_{f},y_{f}z_{f},R_{x},R_{y},R_{z},F_{x},F_{y},F_{z}\right\}$$

In the shearer motion planning task, the action space consists of the desired angle changes q of the two rocker arms and the increase in the traction force f of the shearer at the next time step. This design allows the algorithm to adjust the angles of the rocker arms and regulate the shearer’s traction force at each decision step, thereby optimizing its operational performance and efficiency:

(6) $$a=\left\{q_{1},q_{2},f,\right\}q_{i}\in \left[-1.25^{{}^{\circ}}\sim 1.25^{{}^{\circ}}\right],f\in \left[0\sim 1\right]$$

The traction force value f is normalized using Unity3D’s physics engine. To avoid excessive angle changes within a single time step, we set an upper limit $\left[-1.25^{{}^{\circ}}\sim 1.25^{{}^{\circ}}\right]$ on the angle changes in the front and rear rocker arms. The initial angle of the rocker arm before the shearer executes an action is defined as {θ_1_, θ_2_, f}. After this time step, the joint angle will be adjusted to {θ_1_ + q_1_, θ_2_ + q_2_, f}. This setup helps prevent instability and unexpected behavior caused by excessive angle changes.

When handling tasks like shearer motion planning, comparing sparse reward functions with traditional demonstration methods and dense rewards allows for a deeper analysis of how different input sources specifically affect the algorithm’s performance.

The reward/penalty value r1 for the distance between the front drum of the shearer and the roof can be expressed as follows:

(7) $$r_{1}=\sqrt{{\left(R_{x}-x_{r}\right)}^{2}+{\left(R_{y}-y_{r}\right)}^{2}+{\left(R_{z}-z_{r}\right)}^{2}}-R$$

The reward/penalty value r1 for the distance between the rear drum and the floor can be expressed as follows:

(8) $$r_{2}=\sqrt{{\left(F_{x}-x_{f}\right)}^{2}+{\left(F_{y}-y_{f}\right)}^{2}+{\left(F_{z}-z_{f}\right)}^{2}}-R$$

The sparse reward is set as follows:

(9) $$r_{3}=\left\{\begin{array}{l} -2,\;\mathrm{Traction}\;\mathrm{force}\;\mathrm{exceeds}\;\mathrm{the}\;\mathrm{traction}\;\mathrm{motor}\;\mathrm{load}\\ -1,\;\mathrm{Front}\;\mathrm{drum}\;\mathrm{collides}\;\mathrm{with}\;\mathrm{the}\;\mathrm{roof}\\ -1,\;\mathrm{Rear}\;\mathrm{drum}\;\mathrm{collides}\;\mathrm{with}\;\mathrm{the}\;\mathrm{floor}\\ 2,\;\mathrm{Traction}\;\mathrm{direction}\;\mathrm{continues}\;\mathrm{to}\;\mathrm{approach}\;\mathrm{the}\;\mathrm{target}\\ 10,\;\mathrm{Task}\;\mathrm{successfully}\;\mathrm{completed} \end{array}\right.$$

The traction direction is determined by whether the shearer’s coordinates are continuously increasing along the coal block sequence.

The total reward function for the task is as follows:

(10) $$r=\frac{100}{C+r_{1}+r_{2}}+r_{3}-\gamma \|a\|$$

**Figure 35:**
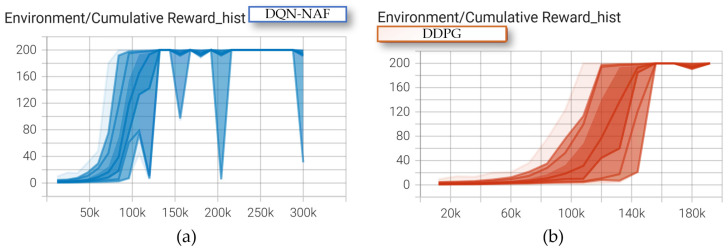
The Environment/Cumulative Reward_hist: (**a**) the DQN-NAF; (**b**) the DDPG.

In this task, both deep reinforcement learning algorithms achieved relatively good results after training the coal mining machine. After exceeding 120,000 training episodes, both algorithms reached convergence for the motion planning task, as shown in Figure 35.

**Figure 36:**
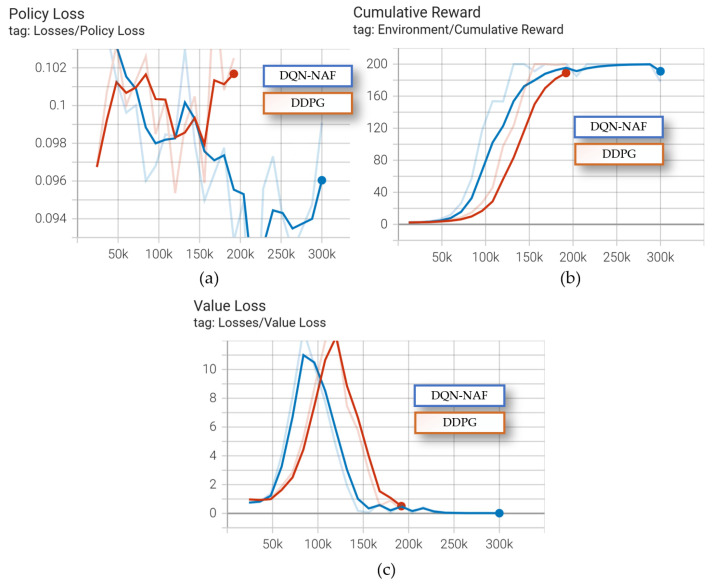
The DQN-NAF and DDPG algorithm performance comparison: (**a**) the policy loss; (**b**) the cumulative reward; (**c**) the value loss.

The cumulative rewards, average loss updates of the value function, and average magnitude of the policy loss function are shown in Figure 36.

As shown in Figure 36, the DQN-NAF algorithm converges faster compared to the DDPG algorithm, as seen in the line graph comparing average episode rewards to the number of episodes. The DDPG algorithm exhibits certain instabilities, requiring multiple iterations to finally converge to satisfactory results. From the comparative results of these two typical algorithms, it can be seen that in the task of coal mining machine planning, the DQN-NAF algorithm demonstrates better performance, while the DDPG algorithm is more unstable and shows larger fluctuations during the training process.

## 6. Conclusions

This paper presents a theory and method for digital twin navigation and cutting planning for coal mining machines, aimed at enhancing the level of intelligence at the coal mining face and achieving the autonomous deduction, learning, and optimization of the shearer. The system has achieved an enhancement from perceptual intelligence to cognitive intelligence. The constructed digital twin navigation and cutting motion planning system provides service support for the shearer cutting digital twin, dynamic navigation map digital twin, reinforcement learning environment construction, and motion planning through a physical perception layer, comprehensive data layer, and data–model integration analysis layer. The experimental results show that within the constructed digital twin environment the deep reinforcement learning DQN-NAF algorithm performs better in terms of effectiveness and stability in shearer motion planning tasks than the DDPG algorithm, effectively enhancing the motion planning capability of the shearer.

There is a gap between reinforcement learning simulations and reality. To translate the outcomes of reinforcement learning into real-world execution, it is necessary to reduce this simulation-to-reality gap through the following approaches: By incorporating more real-world factors, such as noise, dynamic changes, and diverse scenarios, the simulation environment can be made closer to reality. Domain adaptation techniques can be employed to enable the algorithm to effectively perform transfer learning between simulation and reality. During actual execution, the algorithm should be allowed to engage in online learning and adjustment, continuously optimizing strategies by acquiring and utilizing real-time feedback data, thus maintaining good performance in the real world.

## Data Availability

The data presented in this study are available on request from the corresponding author.
